# A meta-analysis of the relationship between social support and physical activity in adolescents: the mediating role of self-efficacy

**DOI:** 10.3389/fpsyg.2023.1305425

**Published:** 2024-01-12

**Authors:** Hao Lin, Haidong Chen, Qingzao Liu, Jie Xu, Shan Li

**Affiliations:** ^1^College of Physical Education, Chengdu University, Chengdu, China; ^2^School of Economics and Management, Shanghai University of Sport, Shanghai, China

**Keywords:** social support, self-efficacy, physical activity, adolescents, meta-analysis

## Abstract

**Introduction:**

Social support influences physical activity (PA) in adolescents. However, whether mediating and moderating effects impact the relationship between them or the underlying mechanism remains unclear. This study aimed to investigate the mediating effects of self-efficacy as well as a series of moderating influences using a meta-analytic approach.

**Methods:**

A total of 56 studies published between January 2001 and May 2023 were obtained from the Web of Science, EBSCO, Taylor and Francis, Scopus, Wiley, ProQuest, and CNKI (core) databases, comprising 65 independent samples (total sample size = 47,196).

**Results and discussion:**

The mean weighted correlation coefficients between social, family, peer, and school support and adolescent PA were 0.298, 0.226, 0.256, and 0.142, respectively, which were significant, except for school support. Family support and teenage PA were related, although the connection was moderated by gender and socioeconomic characteristics. While social, family, and peer support had a direct influence on adolescent PA, school support did not. Self-efficacy mediated the relationship between social support and its subtypes and adolescent PA.

## 1 Introduction

Physical activity (PA) in adolescents has numerous physical and mental health benefits, such as curbing overweight and obesity, improving the cardiovascular system, promoting psychological wellbeing, and enhancing their sense of personal responsibility and group participation. On the contrary, insufficient PA, which is a serious global burden, increases the risk of developing non-communicable diseases (Janssen and LeBlanc, 2010; Hills et al., 2015) and has significantly increased owing to various reasons such as rapid advancements in information technology and drastic lifestyle changes (Aubert et al., 2018). Approximately 80% of adolescents worldwide do not meet the recommended level of activity by the World Health Organization—“at least 1 h of physical activity per day”—further highlighting the importance of investigating the factors impacting the PA levels in this population. It is noteworthy that young individuals’ active participation in sports not only involves intrinsic motivation but also extrinsic support. Social support, a key social resource that aims to help adolescents achieve their desired goals, has become a stable factor in predicting adolescents’ PA behavior in the current research (Beets et al., 2006).

In recent years, many scholars have conducted empirical studies to explore the mechanism underlying the influence of external factors, such as social support and its sub-dimensions, on youth sports activities based on the social support theory. Khan and Uddin (2020) found that parental and peer support were significantly and positively associated with PA levels in Malaysian adolescents. Other scholars investigated the mediating effects of factors such as self-efficacy, subjective exercise experience, exercise commitment, and mental toughness on the association between social support and PA. In particular, the concept of self-efficacy, which refers to an individual’s belief in their own capability to successfully execute a certain action necessary for attaining a desired result has frequently been employed as a mediating variable to predict the PA level. Nevertheless, conclusions regarding the strength of the overall relationship between social support and its sub-dimensions and youth PA and the extent to which self-efficacy mediates their relationship remain unclear. Previous meta-analysis studies have explored the relationship between parental support and youth PA only from a simple perspective (Yao and Rhodes, 2015) but did not compare social support and its subtypes as a whole. In addition, they focused on integrating the effects of the studies and not on the possible mediating effects (Laird et al., 2016). Therefore, in the present study, we drew on Bronfenbrenner’s (1986) ecological theory and classified social support into three subtypes—family, peer, and school support—to explore the correlations and potential moderators of the association between social support and its subtypes and adolescent PA using meta-analytic methods and to validate the mediating role of self-efficacy between them. We believe our findings could provide theoretical and practical support for promoting PA among adolescents.

### 1.1 Relationship between social support and PA

Social support refers to the assistance provided to an individual by significant others, such as family members, friends, peers, relatives, and neighbors, which could be in the form of socioemotional help, practical support, or informational aid, among others (Thoits, 1995). Based on different insights into social support, this form of support can be provided at two levels: (1) at the source level (family, peer, and significant other support) (Zimet et al., 1988) and (2) at the type level (emotional, instrumental, and informational support) (García-Martín et al., 2016).

Social support in PA is a behavior that is intended to help others reach their desired PA goals (Prochaska et al., 2002). Adolescents who perceive and comprehend sources of social support, comprising indirect verbal and non-verbal support, as well as direct tangible assistance, tend to develop positive, stable, and enduring strength to promote emotional engagement in sports participation, thus positively influencing their PA level (Sallis et al., 2000). A considerable number of studies (Beets et al., 2010; Fitzgerald et al., 2012) suggested that social support and its sub-variables are positively correlated with adolescent PA and the important predictors of PA. In addition, the studies found that adequate social support is an effective way to promote PA participation among adolescents. However, the literature search aimed to find divergent results. For instance, a study including African-American girls found that a sense of parental support did not correlate with PA levels in girls (Adkins et al., 2004). In addition, when the sources of social support were analyzed separately, studies such as Beets et al. (2006) indicated that supportive behaviors toward PA from peers and parents were the main sources of support for adolescents, with peer support being more influential. On the contrary, other studies suggested that support from parents was more influential than support from peers (Laird et al., 2016). Owing to these conflicting findings, further research is warranted.

### 1.2 Mediating function of self-efficacy

Self-efficacy refers to individuals’ beliefs and assessments of their capability to successfully engage in a particular behavior. People with high self-efficacy tend to aim higher and devote more effort to achieving their goals. It is a core element that directly or indirectly contributes to health behaviors (Bandura, 2004). The correlation between self-efficacy and PA behaviors has been applied to different groups, with some studies confirming a positive correlation between them (Kang et al., 2019). Self-efficacy is also considered to have a stronger influence on adolescent PA compared to other sociocognitive determinants (Rovniak et al., 2002).

Social support is an essential variable in promoting the development of self-efficacy in individuals (Yang et al., 2010). It can help individuals to have more successful experiences, thereby enhancing their self-efficacy (Chen et al., 2020). Feltz and Magyar’s (2006) study suggested that adolescents’ self-efficacy to participate in PA is influenced by a variety of factors, with social support being one of the most powerful influences. Sound social support can increase an individual’s confidence in engaging in PA and improve exercise self-efficacy. The mediating effect of self-efficacy as a common mediating variable (Silva et al., 2014) on the relationship between social support and PA has been investigated in many studies (Zhang et al., 2022; Li et al., 2023). However, there has been no meta-analytical approach to use self-efficacy as a mediating variable to comprehensively reveal the mechanisms by which social support and its subtypes influence adolescent PA, rendering such a study necessary.

### 1.3 Relevant moderating variables

**Figure 1:**
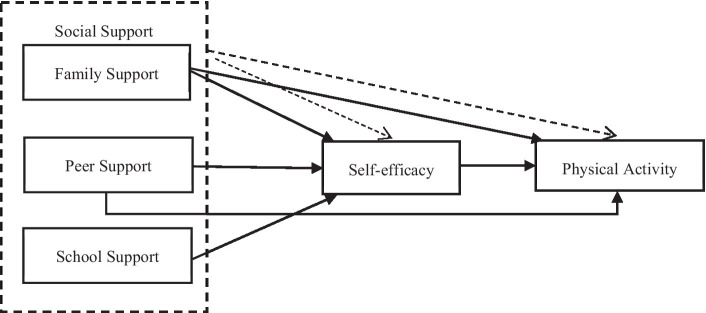
Correlation of variables.

In some meta-analytic studies, situational and individual factors are considered the main moderating variables. Various factors including the adolescent’s grade level, cultural background, socioeconomic status of their residential area, and gender can potentially impact the strength of the relationship between social support and PA. First, social support affects adolescents of different ages differently. For instance, college students are more independent than primary and secondary school students, their parents’ influence gradually fades, and peers become a critical factor influencing their behavior. Second, the function of social support may also vary across cultures. Western cultures are more individualistic, whereas Eastern cultures are more collectivistic. Individuals in collectivistic cultures tend to perceive themselves as interdependent with the group, making them more susceptible to the influences of their significant others than in individualistic countries (Mora and Gil, 2013). Third, PA is related to the stage of economic development. Residents of developed economies are usually more motivated to participate in PA and tend to show higher levels of support for youth PA behaviors than those in developing economies. Finally, there are differences in the type of support each gender wants from their significant others, such as parents. A study found that, while verbal praise is essential for female participants to be physically active, male participants crave co-participation from their parents or peers (Morrissey et al., 2012). Examining the moderating effects of indicators such as academic year, gender, economic level, and cultural background can further help comprehend the underlying mechanisms of the influence of social support on youth PA. The relationship between the variables is shown in Figure 1.

## 2 Methods

### 2.1 Literature search strategy

**Figure 2:**
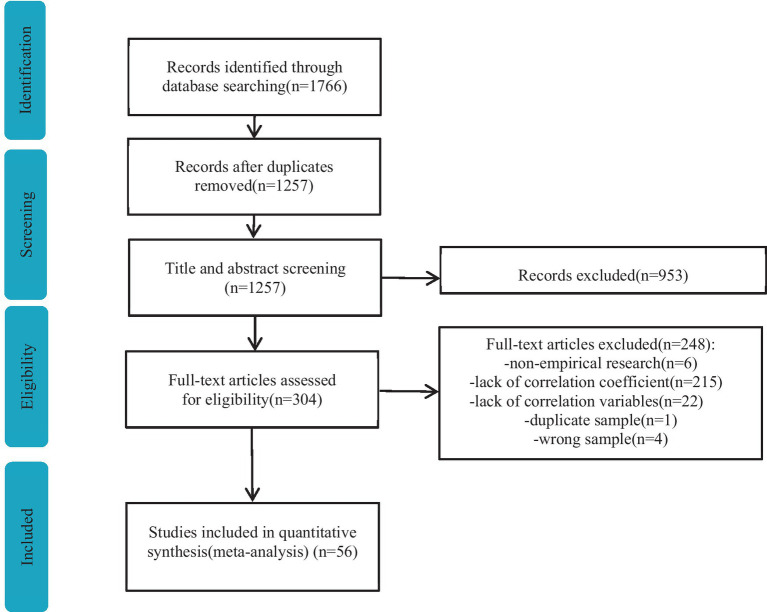
Flowchart of the study selection process.

Databases including the Web of Science, EBSCO, Taylor and Francis, Scopus, Wiley, and ProQuest and CNKI (Core) databases were systematically searched for English language journal articles and dissertations and for Chinese language journal articles, respectively, published between January 2001 and May 2023. A total of 1,766 studies were retrieved (Figure 2).

### 2.2 Criteria for literature screening

The inclusion criteria were as follows: (1) published empirical research journal articles or dissertations; (2) literature that covered all three variables simultaneously: predictor (social support and any of its subtypes: family [mainly parents], peer [classmates and friends], and school [school and teachers] support), mediator (self-efficacy), and outcome variables (PA); (3) literature with reported sample sizes and correlation coefficients *r* or other indicators of transformable data; (4) literature including healthy adolescents from primary school to university level, excluding special groups; (5) if the literature used the same set of data repeatedly, the one with the most accurate data was considered; and (6) cohort studies conducted using baseline data, excluding literature for which baseline data could not be extracted.

### 2.3 Variable coding

The literature included in the study was subjected to coding, which encompassed several aspects such as the initial author and year of publication, sample size, grade level, economic level, cultural background, nature of the literature, and variable relationship. The grade level was divided into primary, secondary, and tertiary levels. The economic level was divided into developed and developing economies according to the International Monetary Fund criteria. However, developing countries with a high gross domestic product *per capita*, such as Saudi Arabia, were excluded. The cultural background was coded as Eastern and Western cultures. Eastern cultures were limited to the East Asian cultural sphere, and Western cultures encompassed countries and regions in Europe, North America, and Oceania. In cases where the literature presented correlation coefficients for social support and its subtypes, the relevant effect values were recorded individually. If the study also covered the three indicator effect values of family, peer, and school support, the average of the three was taken as the social support effect size for statistical purposes. If a study contained two or more independent samples that met the criteria, the samples were coded separately. Two researchers independently extracted the information and completed the coding process, and in the event of a disagreement or inconsistency, a third researcher was involved to reach a consensus. The basic characteristics of the included literature are shown in Table 1.

**Table 1:** Characteristics of the included studies.

Study name	*N*	Grade level	Gender	Economic level/Culture	Publication type	Variable relation
Bajamal et al. (2017)	383	Middle school	NA	NA	Journal	SS_1_&SE&PA
Bao et al. (2022)	4,744	NA	NA	Developing/Eastern	Journal	PS&SE&PA
Bean (2007)	57	Primary school	NA	Developed/Western	Doctoral dissertation	SS_1_&SE&PA
Blake et al. (2017)	361	College	NA	Developed/Western	Journal	SS_1_&SE&PA
Bridges-Arzaga (2012)	299	College	NA	Developed/Western	Master dissertation	FS/PS&SE&PA
Chen et al. (2017)	409	Middle school	NA	Developing/Eastern	Journal	PS&SE&PA
Cheng (2019)	1,188	College	NA	Developing/ Eastern	Journal	PS&SE&PA
Deng (2019)	460	College	NA	Developing/ Eastern	Doctoral dissertation	FS/PS&SE&PA
Dewar et al. (2013)	235	Middle school	F	Developed/Western	Journal	FS&SE&PA
Dishman et al. (2010)	971	Middle school	F	Developed/Western	Journal	SS_1_&SE&PA
Dong et al. (2018)	1,402	Middle school	NA	Developing/Eastern	Journal	SS_1_&SE&PA
Engels et al. (2022)	357	Middle school	NA	Developed/Western	Journal	FS/PS&SE&PA
Farren et al. (2017)	184	College	F	Developed/Western	Journal	SS_1_&SE&PA
Farren et al. (2017)	212	College	M	Developed/Western	Journal	SS_1_&SE&PA
Farren (2014)	212	College	M	Developed/Western	Journal	SS_1_&SE&PA
Farren (2014)	184	College	F	Developed/Western	Master dissertation	SS_1_&SE&PA
Gao (2012)	120	Primary school	NA	Developed/Western	Journal	SS_1_&SE&PA
Hamilton et al. (2017)	226	Middle school	NA	Developed/Western	Journal	PS&SE&PA
Heitzler et al. (2010)	720	NA	NA	Developed/Western	Journal	FS/PS&SE&PA
Jekauc et al. (2015)	101	College	NA	Developed/Western	Journal	FS/PS&SE&PA
Harris (2022)	11	NA	F	Developed/Western	Master dissertation	SS_1_&SE&PA
Kim et al. (2015)	108	College	NA	Developed/Eastern	Journal	FS/PS&SE&PA
Kim and Cardinal (2010)	1,347	Middle school	NA	Developed/Eastern	Journal	FS/PS&SE&PA
Kiyani et al. (2021)	618	NA	NA	Developing	Journal	SS_1/_FS/PS/SS_2_&SE&PA
Lee et al. (2016)	302	NA	NA	Developed/Eastern	Journal	FS&SE&PA
Li et al. (2023)	985	College	NA	Developing/Eastern	Journal	SS_1_&SE&PA
Li et al. (2023)	1,076	College	NA	Developing/Eastern	Journal	SS_1_&SE&PA
Lubans et al. (2012)	1,164	Middle school	F	Developed/Western	Journal	SS_1_&SE&PA
Maglione and Hayman (2009)	85	College	NA	Developed/Western	Journal	SS_1_&SE&PA
Marr and Wilcox (2015)	838	College	NA	Developed/Western	Journal	SS_1_&SE&PA
Martin et al. (2011)	509	Middle school	NA	Developed/Western	Journal	PS&SE&PA
Marttinen et al. (2021)	78	Primary school	NA	Developed/Western	Journal	FS&SE&PA
Mohamadian and Ghannaee Arani (2014)	495	Middle school	NA	Developing	Journal	SS_1_&SE&PA
Morrissey et al. (2012)	147	NA	M	Developed/Western	Journal	SS_1/_FS/PS/SS_2_&SE&PA
Morrissey et al. (2012)	144	NA	F	Developed/Western	Journal	SS_1/_FS/PS/SS_2_&SE&PA
Motl et al. (2007)	1,655	Middle school	F	Developed/Western	Journal	SS_1_&SE&PA
Niermann et al. (2022)	283	Primary school	NA	Developed/Western	Journal	FS&SE&PA
Peterson (2013)	652	NA	M	Developed/Western	Doctoral dissertation	SS_1_&SE&PA
Peterson (2013)	769	NA	F	Developed/Western	Doctoral dissertation	SS_1_&SE&PA
Petosa et al. (2003)	350	College	NA	Developed/Western	Journal	FS/PS&SE&PA
Wenthe (2007)	102	NA	M	Developed/Western	Doctoral dissertation	FS/PS&SE&PA
Wenthe (2007)	103	NA	F	Developed/Western	Doctoral dissertation	FS/PS&SE&PA
Qu et al. (2015)	1,436	Middle school	NA	Developing/Eastern	Journal	SS_1_&SE&PA
Ramirez et al. (2012)	479	Primary school	NA	Developed/Western	Journal	SS_1_&SE&PA
Ren et al. (2020)	1,178	Middle school	M	Developing/Eastern	Journal	SS_1_&SE&PA
Ren et al. (2020)	1,163	Middle school	F	Developing/Eastern	Journal	SS_1_&SE&PA
Sheng et al. (2023)	336	College	NA	Developing/Eastern	Journal	PS&SE&PA
Silva et al. (2014)	203	Middle school	NA	Developed/Western	Journal	FS/PS&SE&PA
Sylvia-Bobiak and Caldwell (2006)	874	College	NA	Developed/Western	Journal	FS/PS&SE&PA
Zhang et al. (2012)	285	Middle school	NA	Developed/Western	Journal	SS_1/_FS/PS/SS_2_&SE&PA
Taymoori et al. (2010)	558	Middle school	F	Developing	Journal	FS/PS&SE&PA
Taymoori et al. (2010)	515	Middle school	M	Developing	Journal	FS/PS&SE&PA
Voskuil et al. (2019)	517	NA	F	Developed/Western	Journal	SS_1_&SE&PA
Wang et al. (2019)	272	Middle school	F	Developed/Western	Journal	FS&SE&PA
Wenthe et al. (2009)	87	NA	M	Developed/Western	Journal	SS_1/_FS/PS/SS_2_&SE&PA
Wenthe et al. (2009)	86	NA	F	Developed/Western	Journal	SS_1/_FS/PS/SS_2_&SE&PA
Wing et al. (2016)	595	Primary school	NA	Developed/Western	Journal	FS&SE&PA
Wu and Pender (2002)	969	Middle school	NA	Developed/Eastern	Journal	SS_1_&SE&PA
Yan (2013)	649	College	NA	Developed/Western	Doctoral dissertation	PS&SE&PA
Yao et al. (2023)	1,510	Middle school	NA	Developing/Eastern	Journal	SS_1_&SE&PA
Yiming et al. (2023)	569	Middle school	NA	Developing/Eastern	Journal	SS_1_&SE&PA
Zhang et al. (2018)	235	College	F	Developed/Western	Journal	FS/PS&SE&PA
Zhang et al. (2022)	1,440	College	NA	Developing/Eastern	Journal	SS_1/_FS/PS/SS_2_&SE&PA
Zhu et al. (2019)	6,394	NA	NA	Developing/Eastern	Journal	FS/PS&SE&PA
Zou et al. (2023)	2,200	Middle school	NA	Developing/Eastern	Journal	PS&SE&PA

N, sample size; NA, not applicable; M, male; F, female; SS1, social support; FS, family support; PS, peer support; SS2, school support; PA, physical activity.

### 2.4 Statistical methods

In this study, the correlation coefficient *r* was selected as the meta-analytic effect size because it is dimensionless and a commonly used statistical indicator of meta-analytic effect sizes in the social science literature (Krasnikov and Jayachandran, 2008). Statistical processing for effect size conversion and merging, heterogeneity testing, publication bias, and moderated effects analysis were performed using CMA 3.7 software. The correlation coefficients *r* extracted from each study were subjected to Fisher’s *Z* transformation according to the methodology recommended by Hedges and Olkin (1985) to calculate a weighted average effect value for each set of relationships. The interconversion formula for *r* and Fisher’s Z is shown:

$$\begin{array}{l} \mathrm{Fishe}r^{\prime }s\;Z=0.5\times \ln\left(\frac{1+r}{1-r}\right),V_{z}=1/\left(n-3\right),\\ \;SE_{z}=\sqrt{V_{z}},r=\left(e^{2z}-1\right)/\left(e^{2z}+1\right)\text{.} \end{array}$$

Heterogeneity across combined effect sizes was assessed by Cochran Q and *I*^2^ statistics (*I*^2^ < 25%, no heterogeneity; 25% ≤ *I*^2^ ≤ 50%, moderate heterogeneity; 50%<*I*^2^<75%, large heterogeneity; and *I*^2^ ≥ 75%, extreme heterogeneity). When the true effect size varies according to differences in the study population and settings, it is recommended to use a random effects model because it is more conservative than a fixed effects model (Cooper et al., 2009; Geyskens et al., 2009). In addition to the point estimates of effect values, 95% confidence intervals (CIs) for the estimated correlation coefficients need to be reported. Publication bias was estimated using three methods. The first is by calculating the fail-safe N to determine the number of additional non-significant studies needed for the overall effect value to become insignificant (Orwin, 1983). According to Rosenthal (1986), when the value of fail-safe N is larger than 5 k + 10 (*k* is the number of studies), then publication bias is not a serious threat, and when it exceeds 100, it is sufficient to prove the absence of publication bias. The second is by drawing a funnel plot; the presence of publication bias is assessed by visually judging the symmetry of the funnel plot. If the funnel plot is symmetrical, it indicates that there is no publication bias. Third, when publication bias is demonstrated by the above two methods, the funnel plot is corrected using the trim-and-fill method to obtain an adjusted effect size that is considered to be closer to the true effect size (Duval and Tweedie, 2000).

Potential moderators were analyzed using mixed-effects models, specifically random-effects models within subgroups and fixed-effects models between subgroups. The Qb values were used to test for significant differences in effect sizes between subgroups of the moderator variables (Kirca et al., 2011).

The mediating effects of path analysis and self-efficacy were validated using webMASEM, a web application for meta-analytic structural equation modeling (MASEM)^1^ developed by Jak and Cheung (2020).

## 3 Results

The search retrieved a total of 1,766 documents, of which 56 (out of 65 independent samples) were ultimately included in the meta-analysis. The total sample size was 47,196, concentrated between the ages of 7 and 30 years, all within the primary-level to tertiary-level student populations. The included studies were mainly conducted in the United States (46.4%) and China (26.8%), followed by Australia, Canada, South Korea, Pakistan, Iran, Saudi Arabia, and Europe.

### 3.1 Homogeneity tests

As shown in Table 2, the test suggested heterogeneity in the results among included studies (*p* < 0.001), further justifying the use of a random-effects model. The *I*^2^ values for all groups reached 75%, indicating a great deal of heterogeneity between effect sizes and that there could be moderating variables that potentially moderated the effect sizes, requiring moderated effect tests.

**Table 2:** Pairwise meta-analysis.

Variable relation	*k*	*r*	95%CI		*z*	*p*-value	*Q_w_*	*df*	*I* ^2^	Fail-safe *N*
			LL	UL						
SS1&PA	35	0.298	0.236	0.359	45.226	0.000	827.438***	34	95.89%	5,398
FS&PA	29	0.226	0.191	0.260	12.416	0.000	116.612***	28	75.99%	4,780
PS&PA	31	0.256	0.226	0.286	16.179	0.000	147.449***	30	79.65%	10,014
SS2&PA	7	0.142	−0.011	0.289	1.817	0.069	76.117***	6	92.12%	152
SS1&SEss1	35	0.374	0.323	0.424	60.690	0.000	627.007***	34	94.58%	6,611
FS&SEfs	29	0.284	0.236	0.330	11.233	0.000	248.371***	28	88.73%	8,367
PS&Seps	31	0.339	0.297	0.380	14.735	0.000	356.230***	30	91.58%	8,475
SS2&SEss2	7	0.149	0.042	0.252	2.726	0.006	35.270***	6	82.99%	74
SEss1&PA	35	0.385	0.305	0.459	60.209	0.000	1527.136***	34	97.77%	7,490
SEfs&PA	29	0.301	0.246	0.355	10.101	0.000	360.746***	28	92.24%	9,443
SEps&PA	31	0.318	0.263	0.370	10.804	0.000	598.745***	30	94.99%	7,430
SEss2&PA	7	0.296	0.198	0.388	5.750	0.000	32.623***	6	81.61%	352

***p < 0.001. CI, confidence interval; SS1, social support; FS, family support; PS, peer support; SS2, school support; PA, physical activity; SEss1, self-efficacy in the social support dimension; SEps, self-efficacy on the peer support dimension; SEss2, self-efficacy on the school support dimension; k, number of studies; r, sample-size weighted mean uncorrected correlation; LL, lower limit; UL, upper limit; Qw, intra-group heterogeneity test statistics.

### 3.2 Publication bias test

The initial visual assessment was performed using a funnel plot (Supplementary material). The results revealed that the majority of the effect values pertaining to the association between social support and its subtypes and adolescent PA were clustered in the upper-middle part. These effect sizes were symmetrically distributed on either side of the total effect size. The validation was performed by calculating the fail-safe N (Table 2), which obtained a critical value of >5 *k* + 10 for each of the items. In addition, no other studies were identified for inclusion through the trim-and-fill method. Therefore, the present meta-analysis could be considered without publication bias.

### 3.3 Main effects test

The average weighted correlation coefficients between social, family, peer, and school support and youth PA were 0.298, 0.226, 0.256, and 0.142, respectively, with *z*-values and value of ps of two-tailed tests reflecting the statistical significance of the point estimates and only school support failing to reach significance (*p* = 0.069 > 0.05). According to the criteria proposed by Cohen (1992), ∣*r*∣ ≤ 0.10 as a small effect value, ∣*r*∣ = 0.30 as a medium effect value, and ∣*r*∣ ≥ 0.50 as a large effect value, the strength of the relationship between social support and its subtypes and youth PA was found to be relatively weak. The mean weighted correlation coefficients between social, family, peer, and school support and self-efficacy were 0.374, 0.284, 0.339, and 0.149, respectively, reaching significance and presenting positive correlations of moderate and low strengths. Furthermore, the average weighted correlation coefficients between self-efficacy and PA in adolescents considering social, family, peer, and school support were 0.385, 0.301, 0.318, and 0.296, respectively. These coefficients were statistically significant and indicated a moderately strong positive correlation.

### 3.4 Moderating effects test

To validate the effects of moderators on the relationship between social support and its subtypes and youth PA, we divided school years into three subgroups (primary, secondary, and tertiary), economic levels into two subgroups (developing and developed economies), and cultures into two subgroups (Eastern and Western). Studies that could not be categorized into subgroup comparisons were excluded, and comparisons were made when three or more study effect sizes were required for each subgroup.

Table 3 shows that social, family, and peer support had a significant positive effect (*p* < 0.05) on adolescent PA, irrespective of their grade level, gender, economic level, and cultural background. Based on the school year factor, the effect size of social support on youth PA gradually increased with advancing grades but decreased for family support. However, none of the differences between the subgroups was significant (*p* > 0.05). Regarding economic factors, the differences only influenced the relationship between family support and youth PA (Qb = 4.197, *p* = 0.04 < 0.05). The effect size was significantly larger in developing than in developed economies (0.274 > 0.207). Cultural factors did not have a significant effect on the relationship between social support and its subtypes and PA (p > 0.05). Finally, the gender factor only moderated the relationship between family support and adolescent PA (Qb = 5.545, *p* = 0.02 < 0.05). The effect size was significantly larger for boys than for girls (0.358 > 0.225).

**Table 3:** Moderator analysis.

	Moderator		*Q_b_*	*df*	*p*	*k*	*r*	95% CI		*z*	*p*
SS1	Grade Level	1	4.511	2	0.105	3	0.216	0.035	0.383	2.332	0.020
		2				13	0.298	0.212	0.380	6.529	0.000
		3				10	0.437	0.300	0.556	5.775	0.000
	Economic Level	4	0.767	1	0.381	11	0.338	0.219	0.448	5.324	0.000
		5				23	0.279	0.212	0.342	7.970	0.000
	Culture	6	0.712	1	0.399	10	0.343	0.217	0.458	5.115	0.000
		7				22	0.282	0.211	0.351	7.522	0.000
	Gender	8	0.825	1	0.364	6	0.355	0.179	0.508	3.834	0.000
		9				12	0.268	0.190	0.343	6.533	0.000
FS	Grade Level	1	1.074	2	0.585	3	0.179	0.094	0.260	4.112	0.000
		2				8	0.214	0.132	0.293	5.037	0.000
		3				8	0.155	0.079	0.230	3.953	0.000
	Economic Level	4	4.197	1	0.040	6	0.274	0.230	0.316	11.829	0.000
		5				23	0.207	0.160	0.254	8.383	0.000
	Culture	6	0.846	1	0.358	6	0.240	0.192	0.287	9.545	0.000
		7				20	0.207	0.154	0.259	7.492	0.000
	Gender	8	5.454	1	0.020	4	0.358	0.298	0.415	10.850	0.000
		9				6	0.225	0.125	0.319	4.378	0.000
PS	Grade Level	2	0.458	1	0.498	10	0.259	0.190	0.325	7.147	0.000
		3				11	0.285	0.249	0.320	14.943	0.000
	Economic Level	4	0.344	1	0.558	11	0.246	0.199	0.291	10.047	0.000
		5				20	0.264	0.223	0.305	12.141	0.000
	Culture	6	0.001	1	0.976	10	0.268	0.230	0.305	13.207	0.000
		7				18	0.267	0.220	0.313	10.709	0.000
	Gender	8	3.372	1	0.066	4	0.352	0.257	0.440	6.892	0.000
		9				5	0.183	0.020	0.336	2.202	0.028

1, primary school; 2, secondary school; 3, college; 4, developing; 5, developed; 6, Eastern; 7, Western; 8, male; 9, female; Q_b_, test statistics for inter-group heterogeneity; *k*, number of studies; *r,* sample-size weighted mean uncorrected correlation; CI, confidence interval; SS1, social support; FS, family support; PS, peer support.

### 3.5 Modeling analysis of elemental analysis structural equations

A mediated effects test for path analysis and self-efficacy based on the MASEM theory was conducted. The joint correlation matrix was first calculated using webMASEM (Table 4), and the results displayed significant positive two-by-two correlation coefficients among social support and its three sub-dimensions (family, peer, and school support), self-efficacy, and PA.

**Table 4:** Correlation matrix for MASEM.

Variable	SS1	SEss1	FS	SEfs	PS	SEps	SS2	SEss2
SEss1	0.364	1.000						
PAss1	0.288	0.363						
SEfs	—	—	0.280	1.000				
PAfs	—	—	0.223	0.292				
SEps	—	—	—	—	0.333	1.000		
PAps	—	—	—	—	0.255	0.309		
SEss2	—	—	—	—	—	—	0.150	1.000
PAss2	—	—	—	—	—	—	0.142	0.292

MASEM, meta-analytic structural equation modeling; SS1, social support; SEss1, self-efficacy in the social support dimension; FS, family support; SEfs, self-efficacy on the family support dimension; PS, peer support; SEps, self-efficacy on the peer support dimension; SS2, school support; SEss2, self-efficacy on the school support dimension; PAss1, physical activity variable for the social support dimension; PAfs, physical activity variable for the family support dimension; PAps, physical activity variable for the peer support dimension; PAss2, physical activity variable for the school support dimension.

Second, the lavaan syntax was composed (using family support as an example: direct effects are # regression coefficients, SE ~ b21*FS, PA ~ b31*FS + b32*SE, and # variances, FS ~ ~ 1*FS, SE ~ ~ p22*SE, PA ~ ~ p33*PA; indirect effects are # regression coefficients, SE ~ beta1*FS, PA ~ beta2*SE + FS, and # variances, FS ~ ~ 1*FS, SE ~ ~ SE, PA ~ ~ PA) for path analysis and mediation effects tests. The fit indices showed that the model was saturated (*χ*^2^ < 0.001,df = 0,CFI = 1.00,TLI = 1.00,RMSEA = 0), indicating that the data fit the model well. As shown in Table 5, the test results suggested that social support could directly predict self-efficacy and adolescent PA, with path coefficients of 0.364 (95% CI: 0.312–0.415) and 0.180 (95% CI: 0.111–0.248), respectively. In addition, it indirectly affected PA through the mediation of self-efficacy, with an indirect effect value of 0.108 (95% CI: 0.079–0.142), accounting for 37.5% of the total effect. Therefore, approximately 38% of the positive relationship presented between social support and adolescent PA was moderated by self-efficacy.

**Table 5:** MASEM.

Path	*k*	a	CI^a^	b	CI^b^	ab	CI^ab^	c	CI^c^	d
SS1-SE-PA	35	0.364	0.312, 0.415	0.298	0.220, 0.375	0.108	0.079, 0.142	0.180	0.111, 0.248	0.288
FS-SE-PA	29	0.280	0.233, 0.325	0.25	0.183, 0.314	0.070	0.050, 0.092	0.153	0.105, 0.201	0.223
PS-SE-PA	31	0.333	0.283, 0.382	0.252	0.189, 0.313	0.084	0.062, 0.109	0.171	0.128, 0.213	0.255
SS2-SE-PA	7	0.150	0.046, 0.255	0.277	0.175, 0.373	0.042	0.013, 0.077	0.101	−0.050, 0.238	0.143

CI, confidence interval; a, effect of predictor variables (SS1/FS/PS/SS2) on the mediator variable (SE); b, effect of the mediator variable (SE) on the outcome variable (PA); ab, indirect effect of the predictor variable on the outcome variable through the mediator variable; c, direct effect of the predictor variable on the outcome variable; d, total effect of the predictor variable on the outcome variable; SS1, social support; SE, self-efficacy; PA, physical activity; FS, family support; PS, peer support; SS2, school support.

Family and peer support had a direct and significant effect on adolescent PA, with path coefficients of 0.153 (95% CI: 0.105–0.201) and 0.171 (95% CI: 0.128–0.213), respectively. In addition, family and peer support had a direct and significant effect on self-efficacy, with path coefficients of 0.280 (95% CI: 0.233–0.325) and 0.333 (95% CI: 0.283–0.382), respectively. Family and peer support also indirectly influenced adolescent PA through the mediation of self-efficacy, with path coefficients of 0.070 (95% CI: 0.050–0.092) and 0.084 (95% CI: 0.062–0.109), accounting for 31.4 and 32.9% of the total effect, respectively.

School support directly predicted self-efficacy, with an effect value of 0.150 (95% CI: 0.046–0.255); however, its direct prediction of adolescent PA was not significant (95% CI: −0.050–0.238) and required mediation through self-efficacy, with an indirect effect value of 0.042 (95% CI: 0.013–0.077).

## 4 Discussion

### 4.1 Relationship between social support and its subtypes and adolescent PA

Our findings suggest that social, peer, and family support significantly and positively correlated with adolescent PA. Social support from parents, classmates, friends, and significant others, as suggested in the social support theory, can positively influence an individual’s psychological and behavioral performance and development (Kerres Malecki and Kilpatrick Demary, 2002). However, the overall indicator of social support had the strongest relationship with adolescent PA, followed by peer and family support, with medium and low strength effect sizes, respectively. Social support and its subtypes have a facilitating effect on adolescent PA.

The effect size of the relationship between social support and adolescent PA was 0.298, indicating a moderately strong positive correlation. However, a meta-analytic study of adolescent girls by Laird et al. (2016) revealed a weak correlation between social support and PA (*r* = 0.237). Taking gender into account, the effect size of the relationship between social support and adolescent female PA obtained in this study was 0.268, in line with the findings of Laird et al. (2016). The overall measure of social support encompasses indicators such as parents, friends, classmates, siblings, teachers, and neighborhoods. However, the relationship between social support and PA was stronger compared to that with other individual indicators, and this difference may be attributed to the way the measurements were made and statistical criteria, among others.

Of the several subtypes of social support, it was found that peer support had the most significant effect value on adolescent PA. According to the social cognitive theory, peer role modeling contributes to the acquisition and occurrence of individual behaviors, including developing an attitude toward PA and decision-making. Peer support, usually in the form of encouragement, appreciation, and joint participation, fulfills an individual’s sense of belonging and helps overcome obstacles in PA participation (Cheng, 2019). On the other hand, family support, mainly from parents, is usually provided in the form of access to sports equipment, transport to sports venues, and reinforcement and encouragement of participation in spare-time sports activities (Voorhees et al., 2005). We found that the relationship between family support and adolescent PA was weaker than between peer support and PA, with an effect size of 0.226, which differs considerably from the meta-analysis results of Yao and Rhodes (2015) (r = 0.38) but similar to Laird et al. (2016) (*r* = 0.19). This difference may be attributed to the age of the study sample. While Yao and Rhodes (2015) included a predominantly younger group of children and adolescents and a large number of preschool children, Laird et al. (2016) included adolescent girls. Established research suggests that, as a child ages, the impact of family diminishes while that of friends and significant others becomes more vital (Collins and Laursen, 2004; Wang and Fu, 2005). Another scholar noted that, while social support in childhood comes mainly from parents, their dependence gradually shifts toward their peers and school as they progress through adolescence (Santrock, 2013). As they grow older, adolescents spend less time with their parents and more time with their peers. Therefore, peers tend to have a greater influence on them than their parents (Larson et al., 1996), a view supported by the moderating effects test for the school year factor in the present study. However, despite the increasing social relevance of peers in adolescence, parents remain an important social factor influencing their children’s participation in PA (Carr and Weigand, 2001).

Furthermore, we found that the relationship between school support and youth PA was not remarkable. Schools play a crucial role in facilitating PA in students by providing them with hardware and software resources, such as qualified teachers and necessary field equipment. Zhang et al. (2012) found that teacher support showed a moderately strong correlation with PA among school children. However, Laird et al. (2016) found no significant correlation between teacher support and students’ PA, which may be related to the low share of teacher support in school support. For instance, Zhang et al. (2022) pointed out that, among the observed variables of school support, venues and facilities were the most influential factors in students’ PA, and the influence of teacher support was relatively weak. Given that only five studies (seven independent samples) were included in the present meta-analysis, the results should be interpreted with caution. Nonetheless, despite the contradiction, the supportive environment created by physical education teachers can have a positive impact (Zhang et al., 2011) on students’ psychological need fulfillment and intrinsic motivation for PA, and teacher support can play a part through other related factors.

### 4.2 Moderating factors

Social, family, and peer support were found to positively influence PA among adolescents, irrespective of their grade level, gender, economic level, and cultural background. Of these factors, family support demonstrated remarkable differences in the gender and economic level factors. However, the differences were not significant across genders in terms of social and peer support. Nevertheless, they have a common trend that the correlation effect values between social, family, and peer support and PA were greater in boys than in girls. This phenomenon may be attributed to the fact that boys tend to engage in sports activities more actively than girls (Sallis et al., 1996; Webber et al., 2008), driving them to actively seek multifaceted support for their sporting activities from society and the important people in their lives. Another reason could be the traditional attitude; boys are often encouraged to participate in sports activities, especially confrontational sports, such as football and basketball, whereas girls are reinforced to be quiet and ladylike (Greendorfer, 1993). This attitude inadvertently prompts parents, peers, and society at large to be more supportive and inclined to support sports activities in boys than in girls.

Demographically, the intensity of the relationship between family support and youth PA was significantly higher in developing than in developed economies, which may be because, in developed economies, the *per capita* resources for sports activities, such as venues and facilities, are relatively abundant (Wei, 2017), and therefore, adolescents have a high degree of accessibility. However, in developing economies, the *per capita* resources for sports are relatively scarce (Lin, 2017), and adolescents require more support from their families to participate in sports activities. Sallis et al. (1996) suggested that socioeconomic status is an important factor that influences students’ PA, with those from higher socioeconomic status families participating in PA more frequently than students from lower-income families due to the latter’s limited access to resources to support PA. This further illustrates the importance of family support in promoting the development of youth PA in underdeveloped regions where sports resources are scarce. Second, in this study, the samples from developing economies were mainly from mainland China, where adolescents are in the critical period of basic cultural knowledge learning, and their spare time is mainly managed and dominated by their parents (Su and Hu, 2017). Moreover, this time is crowded with extracurricular homework, tutorials, and study counseling (Yang, 2020). Hence, parental support for participation in PA becomes crucial.

### 4.3 Mediating role of self-efficacy

Social support and its subtypes significantly and positively correlated with self-efficacy and youth PA, providing further evidence of the applicability of social support theory and self-efficacy theory to research in the area of adolescent PA (Schwarzer and Renner, 2000). Strong emotional and material support from family, school, and peers can help young individuals to effectively regulate their emotions, improve their attitudes, and increase their ability to overcome adversities in PA (Schaefer et al., 1981; Hagger et al., 2003). Consequently, this emotional and material support will increase their self-confidence in PA participation. Reportedly, adolescents with high self-efficacy are more physically active for extended time periods, at a more reasonable intensity, and with more regular behaviors and greater autonomy (Dong et al., 2013). The mediating effect of self-efficacy between social support and its subtypes and adolescent PA accounted for >30% of the total effect, suggesting that adolescents’ PA was not only influenced by social support and its subtypes but more so by self-efficacy. The results of our study reflect Dong and Mao (2018) view that “adolescents who possess both PA self-efficacy and positive help, support, and attention from the outside world tend to be more effectively motivated to engage in PA behaviors. However, when they fail to obtain sufficient social support, their positive psychology toward PA is suppressed until their behavioral tendencies subside.” Therefore, providing adolescents with sufficient support for PA and continuously improving their PA self-efficacy are effective ways to promote their PA behavior.

The direct effect of self-efficacy on youth PA was stronger compared to that of social support and its subtypes, representing a higher proportion of the mechanisms by which social support and its subtypes influence youth PA. Self-efficacy, which is at the heart of social cognition and, to some extent, reflects adolescents’ level of commitment to exercise (Dong and Mao, 2018), is a major determinant of the promotion of PA and other healthy behaviors (Anderson et al., 2006), a perspective demonstrated in the present study.

In addition, the direct effect of school support on adolescent PA was not significant, and self-efficacy was a fully mediated effect between the two, which may be because students in the school environment are usually under immense pressure to learn, lack emotional self-regulation, and are prone to rebelliousness, thus making them reluctant to accept support and help from schools and teachers (Chen et al., 2019). However, school support could still indirectly promote youth PA by addressing factors such as psychological demand fulfillment and intrinsic motivation for PA.

## 5 Implications

This meta-analytic study constructed a relationship model using self-efficacy as a mediating variable based on the ecosystem theory and in conjunction with the social learning theory. It addressed the controversies between the individual studies and clarified the intrinsic mechanism underlying the relationship between social support and adolescent PA, which was validated through the MASEM technique. In addition, the mediating effects of path analysis and self-efficacy were validated through the MASEM approach using webMASEM (Jak and Cheung, 2020), which is a new attempt of this APP in the field of research in sports psychology. The results of this study clarified the relationship between social, family, school, and peer support and adolescent PA, thus providing a foundation and a new perspective for exploring educational paths to effectively improve students’ PA levels and promote their physical and mental health.

This study has certain practical implications for promoting PA among adolescents. First, it is recommended that, while providing resource support for adolescent PA, all sectors of society should focus on improving PA self-efficacy and guarantee the complete utilization of social support resources. Second, when formulating strategies to improve PA among adolescents, it is important to focus on peer support factors, such as cultivating positive and healthy friendships, building a platform for group PA, and encouraging peers to motivate each other to participate in PA. In addition, parents and society should be aware of gender differences in adolescent PA behaviors and, in particular, provide individualized support based on the differences in the propensity of boys and girls to engage in types of PA.

## 6 Limitations

This study has some limitations. First, this study required the inclusion of literature that contained predictors (at least one of social, family, peer, and school support), mediators (self-efficacy), and outcome variables (PA), leaving the four predictor variables difficult to include in an equal amount of literature. Moreover, the amount of school support literature was extremely limited to allow further moderating factor analyses, which can have an impact on the accuracy of the effect sizes. Second, this study incorporated four moderating variables that were selected for this study in conjunction with the included literature. Nevertheless, following the analysis, a high degree of heterogeneity among the studies remained, which reduced the precision and reliability of the results of the meta-analysis. In future, as the number of studies increases, potential moderators, such as instrumental factors for measuring PA and social support, and type of PA should be included to reduce the impact of heterogeneity on the findings. Third, this study only considered the mediating role of a single variable of self-efficacy while ignoring other cognitive factors that may have an impact on PA. To more fully understand the mechanism underlying the influence of social support on PA, variables such as mental toughness and sports experience should be introduced in the future to explore the possible chained mediating role.

## 7 Conclusion

The overall indicators of social support, peer support, and family support showed a significant positive correlation with adolescent PA, with an effect value showing low-to-moderate correlation. Of these indicators, social support had the strongest correlation with adolescent PA, followed by peer and family support. Gender and economic level factors could moderate the relationship between family support and adolescent PA. While social support, family support, and peer support directly influenced adolescents, school support did not, and self-efficacy played a mediating role between them.

## Data availability statement

The original contributions presented in the study are included in the article/Supplementary material, further inquiries can be directed to the corresponding author.

## Author contributions

HL: Writing – original draft, Writing – review & editing. HC: Writing – original draft. QL: Writing – review & editing. JX: Writing – review & editing. SL: Writing – review & editing.

## Footnotes

^1^https://sjak.shinyapps.io/webMASEM/
