# Supplementary material for: A meta-analysis of the relationship between social support and physical activity in adolescents: the mediating role of self-efficacy
Source: Front Psychol. 2024 Jan 12;14:1305425. doi: 10.3389/fpsyg.2023.1305425 (PMC10811609; doi:10.3389/fpsyg.2023.1305425)
Supplement: Supplementary file 1 [file Data_Sheet_1.zip › Supplementary Material/Supplementary Material.docx]

Supplementary Material

A Meta-Analysis of the Relationship Between Social Support and Physical Activity in Adolescents: The Mediating Role of Self-Efficacy

Hao Lin^1*^, Haidong Chen^1^, Qingzao Liu^1,2^，Jie Xu^1^ and Shan Li^1^

*** Correspondence:**

linh2621@126.com

# Search strategies

## English search strategy(Take the Web of Science for example)

Set#1: TS=(family support) OR TS=(social support) OR TS=(teacher support) OR TS=(friend support) OR TS=(peer support) OR TS=(parental support) OR TS=(school support)

Set#2:TS=(exercis*) OR TS=(physical activit*) OR TS=(sport*)

Set#3:TS=(children) OR TS=(adolescen*) OR TS=(teenager*) OR TS=(student*) OR TS=(youth) OR TS=(Young people)

Set#4:TS=(self-efficacy) OR TS=(self-efficiency)

Set#5:(#1 AND #2 AND #3 AND #4)

All sets:

Indexes = SCI-EXPANDED, SSCI, CPCI-S, CPCI-SSH.

Timespan=2001.01.01-2023.05.31

Language=English

## Chinese search strategy(CNKI)

Set#1: SU=(家长支持 OR 社会支持 OR 家庭支持 OR 父母支持 OR 朋友支持 OR 同伴支持 OR 教师支持 OR 学校支持)

Set#2: SU=(运动 OR 体育活动 OR 身体活动 OR 体育锻炼 OR 体力活动)

Set#3: SU=(青少年 OR 学生 OR 初中生 OR 高中生 OR 儿童)

Set#4: SU=自我效能

Set#5: (#1 AND #2 AND #3AND #4 )

All sets:

Indexes = 北大核心.

Timespan=2001.01.01-2023.05.

# Table S1. Characteristics of the included studies

| Study name | *N* | Grade level | Gender | Economic level/Culture | Publication type | Variable relation |
| --- | --- | --- | --- | --- | --- | --- |
| **Bajamal et al. (2017)** | 383 | Middle school | NA | NA | NURSING RESEARCH(SCIE,SSCI) | SS1&SE&PA |
| **Bao et al. (2022)** | 4744 | NA | NA | Developing/Eastern | CHINA SPORT SCIENCE AND TECHNOLOGY(CSSCI) | PS&SE&PA |
| **Bean (2007)** | 57 | Primary school | NA | Developed/Western | Virginia Commonwealth University(Doctoral dissertation) | SS1&SE&PA |
| **Blake et al. 2017)** | 361 | College | NA | Developed/Western | JOURNAL OF ADVANCED NURSING(SCIE,SSCI) | SS1&SE&PA |
| **Bridges-Arzaga (2012)** | 299 | College | NA | Developed/Western | University of Texas System  (Master dissertation) | FS/PS&SE&PA |
| **Chen et al. (2017)** | 409 | Middle school | NA | Developing/Eastern | JOURNAL OF PEDIATRIC PSYCHOLOGY(SSCI) | PS&SE&PA |
| **Cheng (2019)** | 1188 | College | NA | Developing/Eastern | Journal of Sports and Science(CSSCI) | PS&SE&PA |
| **Deng (2019)** | 460 | College | NA | Developing/Eastern | University of Idaho  (Doctoral dissertation) | FS/PS&SE&PA |
| **Dewar et al. (2013)** | 235 | Middle school | F | Developed/Western | RESEARCH QUARTERLY FOR EXERCISE AND SPORT(SCIE,SSCI) | FS&SE&PA |
| **Dishman et al. (2010)** | 971 | Middle school | F | Developed/Western | JOURNAL OF PEDIATRIC PSYCHOLOGY(SSCI) | SS1&SE&PA |
| **Dong et al. (2018)** | 1402 | Middle school | NA | Developing/Eastern | Journal of Shandong Sport University | SS1&SE&PA |
| **Engels et al. (2022)** | 357 | Middle school | NA | Developed/Western | JOURNAL OF BEHAVIORAL MEDICINE(SSCI) | FS/PS&SE&PA |
| **Farren et al. (2017)a** | 184 | College | F | Developed/Western | JOURNAL OF AMERICAN COLLEGE HEALTH(SSCI) | SS1&SE&PA |
| **Farren et al. (2017)b** | 212 | College | M | Developed/Western | OURNAL OF AMERICAN COLLEGE HEALTH(SSCI) | SS1&SE&PA |
| **Farren (2014)a** | 212 | College | M | Developed/Western | University of North Texas  (Master dissertation) | SS1&SE&PA |
| **Farren (2014)b** | 184 | College | F | Developed/Western | University of North Texas  (Master dissertation) | SS1&SE&PA |
| **Gao (2012)** | 120 | Primary school | NA | Developed/Western | PSYCHOLOGY HEALTH & MEDICINE(SCIE,SSCI) | SS1&SE&PA |
| **Hamilton et al. (2017)** | 226 | Middle school | NA | Developed/Western | HEALTH EDUCATION & BEHAVIOR(SSCI) | PS&SE&PA |
| **Heitzler et al. (2010)** | 720 | NA | NA | Developed/Western | AMERICAN JOURNAL OF HEALTH BEHAVIOR(SSCI) | FS/PS&SE&PA |
| **Jekauc et al. (2015)** | 101 | College | NA | Developed/Western | FRONTIERS IN PSYCHOLOGY  (SSCI) | FS/PS&SE&PA |
| **Jr. Harris (2022)** | 11 | NA | F | Developed/Western | University of Minnesota  (Master dissertation) | SS1&SE&PA |
| **Kim et al. (2015)** | 108 | College | NA | Developed/Eastern | PUBLIC HEALTH NURSING  (SCIE,SSCI) | FS/PS&SE&PA |
| **Kim and Cardinal (2010)** | 1347 | Middle school | NA | Developed/Eastern | JOURNAL OF EXERCISE SCIENCE & FITNESS(SCIE) | FS/PS&SE&PA |
| **Kiyani et al. (2021)** | 618 | NA | NA | Developing | INTERNATIONAL JOURNAL OF ENVIRONMENTAL RESEARCH AND PUBLIC HEALTH(SCIE) | SS1/FS/PS/SS2&SE&PA |
| **Lee et al. (2016)** | 302 | NA | NA | Developed/Eastern | MEDICINE AND SCIENCE IN SPORTS AND EXERCISE | FS&SE&PA |
| **Li et al. (2023)a** | 985 | College | NA | Developing/Eastern | FRONTIERS IN PSYCHOLOGY  (SSCI) | SS1&SE&PA |
| **Li et al. (2023)b** | 1076 | College | NA | Developing/Eastern | FRONTIERS IN PSYCHOLOGY  (SSCI) | SS1&SE&PA |
| **Lubans et al. (2012)** | 1164 | Middle school | F | Developed/Western | HEALTH EDUCATION RESEARCH(SSCI) | SS1&SE&PA |
| **Maglione and Hayman (2009)** | 85 | College | NA | Developed/Western | RESEARCH IN NURSING & HEALTH(SCIE,SSCI) | SS1&SE&PA |
| **Marr and Wilcox (2015)** | 838 | College | NA | Developed/Western | American Journal of Health Education(ESCI) | SS1&SE&PA |
| **Martin et al. (2011)** | 509 | Middle school | NA | Developed/Western | RESEARCH QUARTERLY FOR EXERCISE AND SPORT(SCIE,SSCI) | PS&SE&PA |
| **Marttinen et al. (2021)** | 78 | Primary school | NA | Developed/Western | Collegium Antropologicum | FS&SE&PA |
| **Mohamadian and Ghannaee Arani (2014)** | 495 | Middle school | NA | Developing | Journal of Preventive Medicine and Public Health | SS1&SE&PA |
| **Morrissey et al. (2012)a** | 147 | NA | M | Developed/Western | PEDIATRIC EXERCISE SCIENCE(SCIE) | SS1/FS/PS/SS2&SE&PA |
| **Morrissey et al. (2012)b** | 144 | NA | F | Developed/Western | PEDIATRIC EXERCISE SCIENCE(SCIE) | SS1/FS/PS/SS2&SE&PA |
| **Motl et al. (2007)** | 1655 | Middle school | F | Developed/Western | JOURNAL OF PEDIATRIC PSYCHOLOGY(SSCI) | SS1&SE&PA |
| **Niermann et al. (2022)** | 283 | Primary school | NA | Developed/Western | JOURNAL OF FAMILY STUDIES(SSCI) | FS&SE&PA |
| **Peterson (2013)a** | 652 | NA | M | Developed/Western | South Carolina  (Doctoral dissertation) | SS1&SE&PA |
| **Peterson (2013)b** | 769 | NA | F | Developed/Western | South Carolina  (Doctoral dissertation) | SS1&SE&PA |
| **Petosa et al. (2003)** | 350 | College | NA | Developed/Western | AMERICAN JOURNAL OF HEALTH BEHAVIOR(SSCI) | FS/PS&SE&PA |
| **Wenthe (2007)a** | 102 | NA | M | Developed/Western | The University of Iowa(Doctoral dissertation) | FS/PS&SE&PA |
| **Wenthe (2007)** | 103 | NA | F | Developed/Western | The University of Iowa(Doctoral dissertation) | FS/PS&SE&PA |
| **Qu et al. (2015)** | 1436 | Middle school | NA | Developing/Eastern | Journal of Wuhan Institute of Physical Education(CSSCI) | SS1&SE&PA |
| **Ramirez et al. (2012)** | 479 | Primary school | NA | Developed/Western | PSYCHOLOGY OF SPORT AND EXERCISE(SCIE,SSCI) | SS1&SE&PA |
| **Ren et al. (2020)a** | 1178 | Middle school | M | Developing/Eastern | Children | SS1&SE&PA |
| **Ren et al. (2020)b** | 1163 | Middle school | F | Developing/Eastern | Children | SS1&SE&PA |
| **Sheng et al. (2023)** | 336 | College | NA | Developing/Eastern | Frontiers in Psychology(SSCI) | PS&SE&PA |
| **Silva et al. (2014)** | 203 | Middle school | NA | Developed/Western | PEDIATRIC EXERCISE SCIENCE(SCIE) | FS/PS&SE&PA |
| **Sylvia-Bobiak and Caldwell (2006)** | 874 | College | NA | Developed/Western | LEISURE SCIENCES(SSCI) | FS/PS&SE&PA |
| **Zhang et al. (2012)** | 285 | Middle school | NA | Developed/Western | JOURNAL OF APPLIED SPORT PSYCHOLOGY(SCIE,SSCI) | SS1/FS/PS/SS2&SE&PA |
| **Taymoori et al. (2010)a** | 558 | Middle school | F | Developing | Health Education and Behavior | FS/PS&SE&PA |
| **Taymoori et al. (2010)b** | 515 | Middle school | M | Developing | Health Education and Behavior | FS/PS&SE&PA |
| **Voskuil et al. (2019)** | 517 | NA | F | Developed/Western | RESEARCH IN NURSING & HEALTH(SCIE,SSCI) | SS1&SE&PA |
| **Wang et al. (2019)** | 272 | Middle school | F | Developed/Western | JMIR MHEALTH AND UHEALTH(SCIE) | FS&SE&PA |
| **Wenthe et al. (2009)a** | 87 | NA | M | Developed/Western | PEDIATRIC EXERCISE SCIENCE(SCIE) | SS1/FS/PS/SS2&SE&PA |
| **Wenthe et al. (2009)b** | 86 | NA | F | Developed/Western | PEDIATRIC EXERCISE SCIENCE(SCIE) | SS1/FS/PS/SS2&SE&PA |
| **Wing et al. (2016)** | 595 | Primary school | NA | Developed/Western | AMERICAN JOURNAL OF HEALTH BEHAVIOR(SSCI) | FS&SE&PA |
| **Wu and Pender (2002)** | 969 | Middle school | NA | Developed/Eastern | RESEARCH IN NURSING & HEALTH(SCIE,SSCI) | SS1&SE&PA |
| **Yan (2013)** | 649 | College | NA | Developed/Western | Oregon State University  (Doctoral dissertation) | PS&SE&PA |
| **Yao et al. (2023)** | 1510 | Middle school | NA | Developing/Eastern | Frontiers in Psychology(SSCI) | SS1&SE&PA |
| **Yiming et al. (2023)** | 569 | Middle school | NA | Developing/Eastern | SUSTAINABILITY(SCIE,SSCI) | SS1&SE&PA |
| **Zhang et al. (2018)** | 235 | College | F | Developed/Western | WOMEN & HEALTH(SSCI) | FS/PS&SE&PA |
| **Zhang et al. (2022)** | 1440 | College | NA | Developing/Eastern | Frontiers in Psychology(SSCI) | SS1/FS/PS/SS2&SE&PA |
| **Zhu et al. (2019)** | 6394 | NA | NA | Developing/Eastern | Chin J Sch Health | FS/PS&SE&PA |
| **Zou et al. (2023)** | 2200 | Middle school | NA | Developing/Eastern | CHILDREN-BASEL(SCIE) | PS&SE&PA |

N,sample size;NA,not applicable;M,Male;F,Female;SS1,Social Support;FS,[Family Support](javascript:;);PS,[Peer Support](javascript:;);SS2,[School Support](javascript:;);PA,Physical Activities.

# Supplemental bibliography – included studies

Bajamal, E., Robbins, L. B., Ling, J. Y., Smith, B., Pfeiffer, K. A., Sharma, D. (2017). Physical activity among female adolescents in jeddah, saudi arabia: a health promotion model-based path analysis. Nurs. Res. 66 (6), 473-482. doi: 10.1097/NNR.0000000000000244

Bao, R., Cai, Y. J., Li, K., Chen, S. T., Dong, B. L., Wang, L. J. (2022). Peer support and physical activity of children and adolescents:the mediating roles of intrinsic motivation and self-efficacy. CHINA SPORT SCIENCE AND TECHNOLOGY 58 (03), 35-42. doi: 10.16470/j.csst.2019192

Bean, M. K. V. O. (2007). Physical activity in elementary school girls: implementation and theory-based evaluation of girls on the run. [Doctoral dissertation]. United States -- Richmond: Virginia Commonwealth University.

Blake, H., Stanulewicz, N., Mcgill, F. (2017). Predictors of physical activity and barriers to exercise in nursing and medical students. J. Adv. Nurs. 73 (4), 917-929. doi: 10.1111/jan.13181

Bridges-Arzaga, A. (2012). Ethinicty and gender as predictors of physical activity and social cognitive determinants. [Doctoral dissertation]. El Paso: University of Texas System.

Chen, H., Sun, H. C., Dai, J. (2017). Peer support and adolescents' physical activity: the mediating roles of self-efficacy and enjoyment. J. Pediatr. Psychol. 42 (5), 569-577. doi: 10.1093/jpepsy/jsw103

Cheng, H. (2019). The path of promoting aerobic physical fitness of undergraduate by friend support: the mediated role of self-efficacy and physical activity. Journal of Sports and Science 40 (04), 114-120. doi: 10.13598/j.issn1004-4590.2019.04.016

Deng, Y. (2019). The relationships between self-efficacy, social support and physical activity in chinese college students. [Doctoral dissertation]. United States -- Moscow: University of Idaho.

Dewar, D. L., Plotnikoff, R. C., Morgan, P. J., Okely, A. D., Costigan, S. A., Lubans, D. R. (2013). Testing social-cognitive theory to explain physical activity change in adolescent girls from low-income communities. Res. Q. Exerc. Sport 84 (4), 483-491. doi: 10.1080/02701367.2013.842454

Dishman, R. K., Dunn, A. L., Sallis, J. F., Vandenberg, R. J., Pratt, C. A. (2010). Social-cognitive correlates of physical activity in a multi-ethnic cohort of middle-school girls: two-year prospective study. J. Pediatr. Psychol. 35 (2), 188-198. doi: 10.1093/jpepsy/jsp042

Dong, B. L., Zhang, H., Zhu, Y. Q., Cheng, Y. F. (2018). Influence of health beliefs,self-efficacy and social support on leisure exercise for adolescents. Journal of Shandong Sport University 34 (05), 106-112. doi: 10.14104/j.cnki.1006-2076.2018.05.018

Engels, E. S., Nigg, C. R., Reimers, A. K. (2022). Predictors of physical activity behavior change based on the current stage of change-an analysis of young people from hawai'i. J. Behav. Med. 45 (1), 38-49. doi: 10.1007/s10865-021-00255-5

Farren, G. L. (2014). Factors related to meeting physical activity guidelines in college students: a social cognitive perspective. [Master Dissertation]. United States -- Texas: University of North Texas.

Farren, G. L., Zhang, T., Martin, S. B., Thomas, K. T. (2017). Factors related to meeting physical activity guidelines in active college students: a social cognitive perspective. J. Am. Coll. Health. 65 (1), 10-21. doi: 10.1080/07448481.2016.1229320

Gao, Z. (2012). Urban latino school children's physical activity correlates and daily physical activity participation: a social cognitive approach. Psychol. Health Med. 17 (5), 542-550. doi: 10.1080/13548506.2011.647699

Hamilton, K., Warner, L. M., Schwarzer, R. (2017). The role of self-efficacy and friend support on adolescent vigorous physical activity. Health Educ. Behav. 44 (1), 175-181. doi: 10.1177/1090198116648266

Heitzler, C. D., Lytle, L. A., Erickson, D. J., Barr-Anderson, D., Sirard, J. R., Story, M. (2010). Evaluating a model of youth physical activity. Am. J. Health Behav. 34 (5), 593-606.

Jekauc, D., Volkle, M., Wagner, M. O., Mess, F., Reiner, M., Renner, B. (2015). Prediction of attendance at fitness center: a comparison between the theory of planned behavior, the social cognitive theory, and the physical activity maintenance theory. Front. Psychol. 6. doi: 10.3389/fpsyg.2015.00121

Jr. Harris, T. (2022). African american fathers’ social support and relationship quality with daughters’ physical activity beliefs and behaviors. [Master Dissertation]. United States -- Minnesota: University of Minnesota.

Kim, G. S., Lee, C. Y., Kim, I. S., Lee, T. H., Cho, E., Lee, H., et al (2015). Dyadic effects of individual and friend on physical activity in college students. Public Health Nurs. 32 (5), 430-439. doi: 10.1111/phn.12176

Kim, Y. H., Cardinal, B. J. (2010). Psychosocial correlates of korean adolescents' physical activity behavior. J. Exerc. Sci. Fit. 8 (2), 97-104. doi: 10.1016/S1728-869X(10)60015-9

Kiyani, T., Kayani, S., Kayani, S., Batool, I., Qi, S., Biasutti, M. (2021). Individual, interpersonal, and organizational factors affecting physical activity of school adolescents in pakistan. INTERNATIONAL JOURNAL OF ENVIRONMENTAL RESEARCH AND PUBLIC HEALTH 18 (13). doi: 10.3390/ijerph18137011

Lee, E. Y., An, K., Jeon, J. Y., Rodgers, W. M., Harber, V. J., Spence, J. C. (2016). Biological maturation and physical activity in south korean adolescent girls. MEDICINE AND SCIENCE IN SPORTS AND EXERCISE 48 (12), 2454-2461. doi: 10.1249/MSS.0000000000001031

Li, N., Zhao, S., Liu, C., Dai, K., Huang, W. (2023). Exploring the relationship between perceived social support and college students’ autonomous fitness behavior: chain mediating effect test. Front. Psychol. 13. doi: 10.3389/fpsyg.2022.1036383

Lubans, D. R., Okely, A. D., Morgan, P. J., Cotton, W., Puglisi, L., Miller, J. (2012). Description and evaluation of a social cognitive model of physical activity behaviour tailored for adolescent girls. Health Educ. Res. 27 (1), 115-128. doi: 10.1093/her/cyr039

Maglione, J. L., Hayman, L. L. (2009). Correlates of physical activity in low income college students. Res. Nurs. Health 32 (6), 634-646. doi: 10.1002/nur.20353

Marr, J., Wilcox, S. (2015). Self-efficacy and social support mediate the relationship between internal health locus of control and health behaviors in college students. Am. J. Health Educ. 46 (3), 122-131. doi: 10.1080/19325037.2015.1023477

Martin, J. J., McCaughtry, N., Flory, S., Murphy, A., Wisdom, K. (2011). Using social cognitive theory to predict physical activity and fitness in underserved middle school children. Res. Q. Exerc. Sport 82 (2), 247-255. doi: 10.5641/027013611X13119541883825

Marttinen, R., McAlister, K. L., Ives, S. T., Battistella, S., Fredrick, R. N., Johnston, K., et al (2021). Fitness, pa, perceived c competence, parental support, and literacy outcomes in the reach after-school sports program. Collegium Antropologicum 45 (3), 225-234. doi: 10.5671/ca.45.3.6

Mohamadian, H., Ghannaee Arani, M. (2014). Factors predicting the physical activity behavior of female adolescents: a test of the health promotion model. Journal of Preventive Medicine and Public Health 47 (1), 64-71. doi: 10.3961/jpmph.2014.47.1.64

Morrissey, J. L., Wenthe, P. J., Letuchy, E. M., Levy, S. M., Janz, K. F. (2012). Specific types of family support and adolescent non-school physical activity levels. Pediatr. Exerc. Sci. 24 (3), 333-346. doi: 10.1123/pes.24.3.333

Motl, R. W., Dishman, R. K., Saunders, R. P., Dowda, M., Pate, R. R. (2007). Perceptions of physical and social environment variables and self-efficacy as correlates of self-reported physical activity among adolescent girls. J. Pediatr. Psychol. 32 (1), 6-12. doi: 10.1093/jpepsy/jsl001

Niermann, C., Wagner, P., Ziegeldorf, A., Wulff, H. (2022). Parents' and children's perception of self-efficacy and parental support are related to children's physical activity: a cross-sectional study of parent-child dyads. J. Fam. Stud. 28 (3), 986-1004. doi: 10.1080/13229400.2020.1773901

Peterson, M. S. (2013). The role of self-efficacy as a mediator between parent social support and physical activity in male and female adolescents. [Doctoral dissertation]. United States -- Columbia: South Carolina.

Petosa, R. L., Suminski, R., Hortz, B. (2003). Predicting vigorous physical activity using social cognitive theory. Am. J. Health Behav. 27 (4), 301-310. doi: 10.5993/AJHB.27.4.2

Qu, L. P., Ou, Z. G., Li, Z. H., Tan, S. J., Wang, J. (2015). Model of physical health promotion for adolescents in china. Journal of Wuhan Institute of Physical Education 49 (09), 69-75. doi: 10.15930/j.cnki.wtxb.2015.09.013

Ramirez, E., Kulinna, P. H., Cothran, D. (2012). Constructs of physical activity behaviour in children: the usefulness of social cognitive theory. Psychol. Sport Exerc. 13 (3), 303-310. doi: 10.1016/j.psychsport.2011.11.007

Ren, Z., Hu, L., Yu, J. J., Yu, Q., Chen, S., Ma, Y., et al (2020). The influence of social support on physical activity in chinese adolescents: the mediating role of exercise self-efficacy. Children 7 (3), 23.

Sheng, J., Gong, L., Zhou, J. (2023). Exercise health belief model mediates the relationship between physical activity and peer support among chinese college students: a cross-sectional survey. Front. Psychol. 14. doi: 10.3389/fpsyg.2023.1103109

Silva, P., Lott, R., Mota, J., Welk, G. (2014). Direct and indirect effects of social support on youth physical activity behavior. Pediatr. Exerc. Sci. 26 (1), 86-94. doi: 10.1123/pes.2012-0207

Sylvia-Bobiak, S., Caldwell, L. L. (2006). Factors related to physically active leisure among college students. Leis. Sci. 28 (1), 73-89. doi: 10.1080/01490400500332728

Taymoori, P., Rhodes, R. E., Berry, T. R. (2010). Application of a social cognitive model in explaining physical activity in iranian female adolescents. Health Educ. Res. 25 (2), 257-267. doi: 10.1093/her/cyn051

Voskuil, V. R., Robbins, L. B., Pierce, S. J. (2019). Predicting physical activity among urban adolescent girls: a test of the health promotion model. Res. Nurs. Health 42 (5), 392-409. doi: 10.1002/nur.21968

Wang, T. T., Ren, M. Y., Shen, Y., Zhu, X. R., Zhang, X., Gao, M., et al (2019). The association among social support, self-efficacy, use of mobile apps, and physical activity: structural equation models with mediating effects. JMIR mHealth uHealth 7 (9). doi: 10.2196/12606

Wenthe, P. J. (2007). The predisposing, reinforcing, and enabling factors associated with physical activity and sedentary behavior in males and females during early adolescence. [Doctoral dissertation]. United States -- Iowa: The University of Iowa.

Wenthe, P. J., Janz, K. F., Levy, S. M. (2009). Gender similarities and differences in factors associated with adolescent moderate-vigorous physical activity. Pediatr. Exerc. Sci. 21 (3), 291-304. doi: 10.1123/pes.21.3.291

Wing, E. K., Belanger, M., Brunet, J. (2016). Linking parental influences and youth participation in physical activity in- and out-of-school: the mediating role of self-efficacy and enjoyment. Am. J. Health Behav. 40 (1), 31-37. doi: 10.5993/AJHB.40.1.4

Wu, T. Y., Pender, N. (2002). Determinants of physical activity among taiwanese adolescents: an application of the health promotion model. Res. Nurs. Health 25 (1), 25-36. doi: 10.1002/nur.10021

Yan, Z. (2013). Physical activity among chinese international students in american higher education: from quantitative and qualitative perspectives. [Doctoral dissertation]. United States -- Corvallis: Oregon State University.

Yao, S. J., Ma, Q. S., Liu, C., Cao, D. W., Lyu, T., Guo, K. L. (2023). The relationship between physical exercise and subjective well-being among chinese junior high school students: a chain mediating model. Front. Psychol. 13. doi: 10.3389/fpsyg.2022.1053252

Yiming, Y., Shi, B., Alghamdi, A. A., Kayani, S., Biasutti, M. (2023). Social support and self-efficacy as mediators between internal locus of control and adolescents' physical activity. Sustainability 15 (7). doi: 10.3390/su15075662

Zhang, T., Dunn, J., Morrow, J., Greenleaf, C. (2018). Ecological analysis of college women's physical activity and health-related quality of life. Women Health 58 (3), 260-277. doi: 10.1080/03630242.2017.1296057

Zhang, T., Solmon, M. A., Gao, Z., Kosma, M. (2012). Promoting school students' physical activity: a social ecological perspective. J. Appl. Sport Psychol. 24 (1), 92-105. doi: 10.1080/10413200.2011.627083

Zhang, Y., Hasibagen, Zhang, C. (2022). The influence of social support on the physical exercise behavior of college students: the mediating role of self-efficacy. Front. Psychol. 13. doi: 10.3389/fpsyg.2022.1037518

Zhu, X. L., Sai, X. Y., Geng, Y. G., Yang, M. Q., Zhao, F. Q. (2019). Health behaviors and psychosocial determinants among primary and middle school students in henan province. Chin J Sch Health 40 (08), 1162-1165. doi: 10.16835/j.cnki.1000-9817.2019.08.012

Zou, Y. X., Liu, S. J., Guo, S. S., Zhao, Q. H., Cai, Y. J. (2023). Peer support and exercise adherence in adolescents: the chain-mediated effects of self-efficacy and self-regulation. Children-Basel 10 (2). doi: 10.3390/children10020401
